# Infant and child health status ahead of implementation of an integrated intervention to improve nutrition and survival: a cross-sectional baseline assessment

**DOI:** 10.1186/s40795-020-00372-5

**Published:** 2020-10-05

**Authors:** Emmanuel Nene Odjidja, Sonia Hakizimana, Ghislaine Gatasi, Jean-Berchmans Masabo, Gildas Irakoze, Heritier Muzungu, Honorine Murorunkwere, Leila Raissa Ngabirano, Mahmoud Elkasabi, Barbora De Courten

**Affiliations:** Village Health Works, BP 1604 Bujumbura, Burundi

**Keywords:** Under-five mortality, Child health, Infant health, Malnutrition, Childhood morbidity, Low birth weight, Implementation research, Formative evaluation

## Abstract

**Background:**

Burundi has one of the poorest child health outcomes in the world. With an acute malnutrition rate of 5% and a chronic malnutrition rate of 56%, under five death is 78 per 1000 live births and 47 children for every 1000 children will live until their first birthday. In response to this grim statistics, Village Health Works, a Burundian-American organisation has invested in an integrated clinical and community intervention model to improve child health outcomes. The aim of this study is to measure and report on child health indicator ahead of implementing this model.

**Methods:**

A cross sectional design was employed, adopting the Demographic Health Survey methodology. We reached out to a sample of 952 households comprising of 2675 birth, in our study area. Mortality data was analysed with R package for mortality computation and other outcomes using SPSS. Principal component analysis was used to classify households into wealth quintiles. Logistic regression was used to assess strength of associations and significance of association was considered at 95% confidence level.

**Results:**

The incidence of low birth weight (LBW) was 6.4% at the study area compared to 10% at the national level with the strongest predictor being malnourished women (OR 1.4 95%CI 1.2–7.2 *p = 0.043*). Fever incidence was higher in the study area (50.5%) in comparison to 39.5% nationally. Consumption of minimum acceptable diet was showed a significant protection against fever (OR 0.64 95%CI 0.41–0.94 *p = 0.042*). Global Acute Malnutrition rate was 7.6% and this significantly reduced with increasing age of child. Under-five mortality rate was 32.1 per 1000 live births and infant mortality was 25.7 per 1000 in the catchment with most deaths happening within the first 28 days of life (57.3%).

**Conclusion:**

Improving child health status is complex, therefore, investing into an integrated intervention for both mother and child could yield best results. Given that most under-five deaths occurred in the neonatal period, implementing integrated clinical and community newborn care interventions are critical.

## Background

Every year, the world experiences 5.4 million deaths of children under five, of which 46.3% occurs during the first month of life and 81.5% is from preventable causes [1]. Whereas global under-five mortality rate has reduced from 93 per 1000 live births to 39 per 1000 in 2017, which of sub-Saharan Africa stand at 76 per 1000, implying that 1 in 13 children will die before reaching the 5th birthday [2]. According to a recent United Nations report, with the increasing child population in high mortality countries in the sub-Saharan Africa, it is estimated that the region will accommodate 60% of all childhood mortalities by 2050 [3]. Within the region, the risk of childhood mortality depends on an array of socio-economic and cultural factors which is distinct from country to country. Children in households of the poorest wealth quintile are two times more likely to die compared to those in the richest quintile [4] and children in rural areas are 1.5 times at risk to die than their cohorts in urban areas [5]. The risk of all under-five deaths is 2.6 times more for children whose mothers have little than primary or no education in comparison to those with mothers who have a minimum of secondary education [1].

Complexities of these sociodemographic factors which influence child survival in sub-Saharan Africa are also mirrored by the inequality in nutritious food distribution resulting in differences in the rates of childhood malnutrition across the region [6–8]. International reports and several individual studies have established the link between all forms of childhood malnutrition and risk of child death in sub-Saharan Africa [9–11], directly accounting for 61% of all deaths [12]. Malnutrition is a combined effect of food access, frequency and quality which is deeply rooted in several socio-economic, cultural and environmental factors of households. Some of the causes of malnutrition are poverty [13], household food insecurity, illiteracy, dwindling farmlands, poor sanitation and climate change along with several other background factors [14]. While most interventions have focused on increasing uptake of important nutrients and childhood supplements, little attention has been given to addressing these underlying issues in an integrated way [15, 16]. Nutrition specific interventions have been mostly implemented in parallel with little integration with existing programs hence had little impact on effectively tackling malnutrition, its root causes and all child mortality. Integrating nutritional interventions does not only reduce the risk of childhood mortality [6], but also has long-term effect on overall childhood development, adult health and later life productivity, economic wellbeing as well as epigenetic changes on future generations [17]. Furthermore, emerging evidence has suggested that integrating these two components of nutrition programming can result in significant improvements in cost-effectiveness, impact, sustainability and efficiency of these programs [17].

With a wasting and stunting rate of 5 and 56% respectively, Burundi has one of highest malnutrition rates in the world [18]. Findings from the recent Demographic and Health Surveys DHS [18] show that 78 children in every 1000 will not celebrate their 5th birthday and 47 children per 1000 will not go past the 1st year of birth.

To address this issue, Village Health Works (VHW), a Burundian-American organisation has made significant investment in an integrated program that combines dignified clinical care with targeted food security, economic development, education and community health interlaced with gender and community engagement [19]. Ahead of implementing this program, this study aims to measure and report infant and child health status as well as unpack different components of this model which is influenced on findings of this study.

## Methods

### Study area

**Fig. 1:**
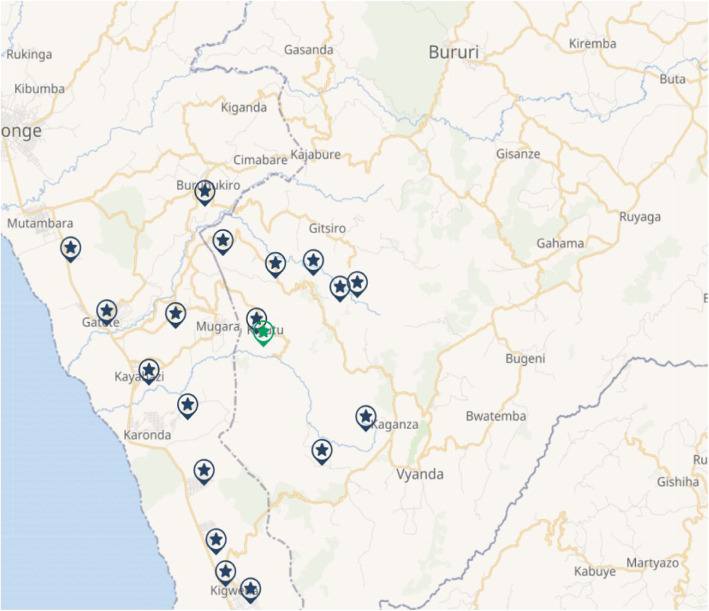
Map of Study Area constructed using ArcGIS mapping software

This prospectively collected data collection was conducted at the Vyanda and Rumonge provinces located in the south of Burundi (Fig. 1). Predetermined *collines* (districts) of VHW’s study area constituted the program target area. However, to avoid outliers in the results, the capital towns of both provinces were excluded. Therefore, the sampling frame of this study constituted 18 *collines* with a total population of 142,953.

Programmatically, at present, VHW is the only organisation working on infant and child health in the area although this coincides with the implementation of the national free healthcare policy for pregnant women and children under-five.

### Sampling strategy

A two-stage sampling strategy comprising of cluster and systematic sampling was used for this evaluation. At the first stage, *collines* along with their respective populations were received from the administrative province authorities. This became the sampling frame from which a probability proportional to size (PPS) was applied to select a desired cluster size of 30.

The second stage was conducted during field work. Enumerators received households list from the chief of *collines* (*chef de la colline*) and depending on the total number of households available in that *colline*, either a 2nd or 3rd consecutive household was systematically selected after a random ‘pen throw’ to select the first household. The main sampling units were households and selection was based on the following inclusion and exclusion criteria:

#### Inclusion criteria

1. Households with women aged 15 and 49 defined in this evaluation as women of reproductive age with children under-five
2. Children under-five with no life-threatening underlying illnesses and capable to undertake anthropometric measurements.
3. Households with women 15–49 who were residents of the area for at least a year
4. Women of reproductive age who were in sound mind to respond on questions relating to childhood fever and birth histories

#### Exclusion criteria

1. Households that did not meet the stated inclusion criteria
2. Households that failed to grant consent via signing or thumb-printing of the form

### Sample size calculation

The determination of an appropriate sample size was based on the methodology of the DHS [18], an international program that conducts national representative surveys on major maternal, infant and child health indicators. Following the statistically robust predetermined conditions of the survey, details of which, have been published elsewhere [18], 952 households were selected. These households then presented 2675 birth histories for computation of infant and child mortality indicators.

### Survey management and data collection

The entire survey was managed by the operational research, monitoring and evaluation department of VHW. Standard questionnaires were adopted from French version of the standard DHS [20] and United Nations Children’s Fund (UNICEF) multiple indicator cluster survey [21]. Questionnaires were segregated into three targeting women (the caregiver in most instances), men and other members of household. Questions about child outcomes were collected from the caregiver and childhood mortality collected through birth histories of women of reproductive age.

Data was collected by seven field teams comprising of a team lead (enumerator), measurer and a supervisor in each team from May 01, 2019 to June 28, 2019. Team leaders interviewed caregivers on child outcomes including taking birth histories and measurers were responsible for taking anthropometric measurements of children and women of reproductive age (height, weight and Mid-Upper Arm Circumference – MUAC). Supervisors ensured data quality and oversaw random selection of households.

Measurements for resting systolic blood pressure (SBP) and diastolic blood pressure (DBP) were done using a digital portable blood pressure monitor (Panasonic, Germany) which were taken for women of reproductive ages at the households. For each study participant, two measurements were taken, 10 min apart and the average was taken as the final reading.

### Definition of outcomes

Main outcomes for assessment in this study were: low birth weight, childhood fever and malnutrition and childhood mortality. To allow comparability of our results with national figures, we defined these outcomes according to standard definitions by the DHS [18].

Low birth weight (LBW) was assessed from birth card of the last pregnancy of women within the reproductive age. We classified low birth weight as children who had a weight below 2500 g at the first measurement after birth.

Fever incidence was defined as child with elevated temperature any level any day within the 2 weeks preceding the survey. This definition is line with the DHS definition and the recall method for data collection has been validated in different settings [22, 23].

Malnutrition was classified under two main measures: acute malnutrition (wasting) and chronic malnutrition (stunting). Global Acute Malnutrition, a combination of moderate and severe wasting was defined as children between 6 and 59 months with weight-for-height (WfH) z-score less than − 2 according to the WHO growth standards and global Chronic Malnutrition on the other hand was defined as children with height-for-age z-score less than − 2 according to the growth standards. A woman was classified as malnourished if she had MUAC ≤23.0 cm to ≤25.5 cm.

From birth and death histories acquired from women and using a synthetic cohort life table approach [24, 25], childhood mortality rates classified as neonatal, post-neonatal, infant, child and under-5 mortality were calculated as:

1. Neonatal mortality rate (NNMR): the probability of a child dying between the first day of birth and exact age equal or less than 1 month;
2. Post-neonatal mortality rate (PNMR): the probability of a child dying between ages after 1 month and equal or less than 1 year. This can also be calculated as the difference between IMR and NNMR;
3. Infant mortality rate (IMR): the probability of a child dying between the first day of birth and equal or less than a year after birth;
4. Child mortality rate (CMR): the probability of a child dying between after 1 year of birth and exact age equal or less than 5 years. This is also calculated as the difference between U5MR and IMR;
5. Under-5 mortality rate (U5MR): the probability of a child dying between the first day of birth and exact age equal or less than 5 years.

### Definition of exposure variables

Minimum Acceptable Diet, Blood Pressure status of caregiver, wealth status of households, Nutritional status of caregiver and Household Hunger Scale of households were included as exposure variables for this study.

Minimum Acceptable Diet (MAD) consumed was defined as the proportion of children who consumed four out of seven food types the day preceding the survey. The food types were; grains, root and tubers, legumes and nuts, dairy products (milk, yoghurt, cheese etc.), flesh food (meat, fish, poultry etc.), eggs, Vitamin A-rich fruits and vegetables and other fruits and vegetables. This information was obtain through recall of food consumed 24 h before the survey.

Blood Pressure (BP) classified was into normal or abnormal (high or low). Normal blood pressure classified as SBP/DBP of 90/60 mmHg and 120/80 mmHg and abnormal blood pressure, < 90/60 mmHg or ≥ 140/90 mmHg.

Household hunger scale, a global indicator for assessing household access and frequency to food was defined as households who reported ‘yes’ to one or more of the following events: 1. no food at all in the house; 2. went to bed hungry, 3. went all day and night without eating. For households that who confirmed ever experiencing any of these events, further questions on frequency were asked which were classified into never (value = 0), rarely or sometimes (value = 1), often (value = 2), summing to a total score of 6. Households with a score between 0 and 1 were classified as ‘No hunger detected in households’, those with score between 2 and 3, ‘Moderate hunger detected in household’ and between scores of 4 and 6, ‘Severe hunger detected in household’.

In assessing if a child had received Vitamin A supplement, a sample was presented to the caregiver and asked if child had received it 6 months preceding the study. When confirmed, the child was considered as having received Vitamin A supplementation.

Wealth quintiles were constructed from principle component analysis of 15 household items, consisting of household possessions, a state of housing and access to essential services. From the component coefficients generated, rank analysis was applied to classify households into five levels with lowest being the poorest and highest being the richest.

Finally, to determine the influence of malnutrition on some outcomes, it was used as an exposure and the definition is same as stated above in outcomes section.

### Data analysis

Mortality rates were computed using version 0.7.0 of a predeveloped R package [24] which was originally developed for calculation of childhood mortality using the DHS methodology [25]. Childhood mortality rates were calculated from birth and death histories acquired from women of reproductive age 60 months (5 years) preceding the survey.

All other indicators were calculated using IBM software – SPSS Statistic version 20 [26]. Chi-square test was used to assess relationships between outcomes and exposure variables (those variables disaggregated by the outcome variables). All outcomes were binary, as such a binary logistic regression was used to assess strength of association. However, when independent variables had more than two variables, a multinomial logistic regression was used. Significance of association was considered at 95% confidence level *p < 0.05* (two-tailed).

Nutrition data was analysed with Emergency Nutrition Assessment (ENA) software [27] for determining the individual malnutrition level of every child (using the WHO defined z-score parameters) and results exported to SPSS for further analysis.

## Results

### General characteristics of respondents and households

Following a response rate of 95.4%, 2675 live births were recorded for mortality computation from birth histories. Of this, 441 (75.9% of all households visited) children under-five were identified and eligible for nutrition screening and 581 women of reproductive age were interviewed. Among these women, 74.8% were married, 21.3% single, 1.1% divorced or separated and 2.0% widowed. Additionally, 0.8% children were identified as orphans and 3.3% reported to have lost either one parent in all households visited. Overall, 74.2% women were involved in paid employment in farming or within the agricultural value chain. Functional literacy was higher among women (56.9%) and 73.8% of all women reported to have ever attended school.

On general characteristics of households where women and children resided, only 6.7% of households had access to electricity. Although 85.4% of households reported to have had access to improved sources of water, 36% reported to have inconsistencies in supply with longer travelling time for water access (22 min). Fifty percent of households had access to improved sanitation services which among those with unimproved sources, majority (50%) reported to using pit latrines with slabs. In determining overall socioeconomic status of households, we employed wealth quintiles of households. Combining the two poorest quintiles (Table 1) show that the study area accommodates 43% compared to only 40% nationally.

**Table 1:** Descriptive characteristics of households in study areas

Indicator	n	%
Functional Literacy among women	331	56.9
Percentage of women ever attended school	429	73.8
Households with access to improved water sources	727	85.4
Households with access to improved sanitation sources	325	50.6
***Wealth Distribution among Households***		
Richest	94	16.2
Fourth	126	21.7
Middle	112	19.2
Second	118	20.3
Poorest	132	22.7

### Low birth weight (LBW)

Overall, the prevalence of low birth weight among eligible women of reproductive age was 31(6.4%) compared to the 10.5% at the national level and 5.8 and 6.8% respectively at Rumonge and Vyanda provinces [18]. Disaggregating the prevalence rate by background information (Table 2), LBW was higher in malnourished women.

**Table 2:** Low Birth Weight Incidence disaggregated by background variables

Variable Disaggregation	n (%)	Odds Ratio (OR)	Sig. Level (***P***-value)
LBW Prevalence	31 (6.4%)	**–**	**–**
LBW Prevalence by **Province**			
*Rumonge*	21 (5.7%)	Ref	0.251
*Vyanda*	10 (8.7%)	0.63	
LBW Prevalence by **BP status of woman**			
*High/Low Blood Pressure*	7(9.1%)	1.8	0.195
*Normal Blood Pressure*	21 (5.3%)	Ref	
LBW Prevalence by **Nutritional Status of woman**			
*Normal Nutritional*	20 (4.8%)	Ref	0.043
*Malnourished*	8 (12.5%)	1.40	
LBW Prevalence by **Household Hunger Scale**			
*No hunger detected in household*	17 (5.0%)	0.32	0.300
*Moderate Hunger detected in household*	13 (9.4%)	0.62	0.673
*Severe Hunger detected in household*	1 (14.3%)	Ref	Ref
LBW Prevalence by **Wealth Quintiles**			
*Richest*	2 (2.0%)	0.30	0.142
*Fourth*	5 (5.2%)	0.78	0.689
*Middle*	9 (8.7%)	1.37	0.56
*Second*	6 (6.5%)	Ref	Ref
*Poorest*	9 (9.7%)	1.54	0.434

All five indicators were used to create a model to predict risk of low birth weight. The model showed that 81% (Nagelkerke R^2^) of the variance in low birth weight and accurately classified 94.1% of all cases. Among the five variables, malnutrition among women were significantly associated with low birth weight (OR 1.4 95% CI 1.2–7.2 *p = 0.*043) (Table 2).

### Incidence of childhood fever

General fever prevalence among children under 5 was 49.5% in comparison to 34.3 and 35.3%, respectively, in Rumonge and Vyanda province with 42% at the national level [18]. Child nutrition was associated with fever incidence and this was evident from an assessment of all indicators on food frequency, access and quality. Household Hunger Scale, an indicator that measures access and frequency of food shows that children in households with severe hunger were more predisposed to fever than those with moderate and no hunger (Table 3). Consumption of acceptable minimum diet, an indicator that measures food quality and diversity consumed by both breastfed and non-breastfed children showed a significant protection against fever (OR 0.64 95%CI 0.41–0.94 *p = 0.042*). As expected, children that did not sleep in insecticide treated net the night before the survey were more likely to develop fever (1.57).

**Table 3:** Prevalence of Fever disaggregated by background variables

Variable Disaggregation	n (%)	Odds Ratio (OR)	Sig. Level (P-value)
Prevalence by **Province**			
*Rumonge*	207 (48.9%)	0.92	0.659
*Vyanda*	67 (51.1%)	Ref	Ref
Prevalence by S**tunting Status of Child**			
*No Stunting*	151 (50.0%)	1.07	0.707
*Stunted*	40 (48.4%)	Ref	Ref
Prevalence by **Household Hunger Scale**			
*No hunger detected in household*	180 (47.5%)	0.20	0.043
*Moderate Hunger detected in household*	85 (51.8%)	0.23	0.050
*Severe Hunger detected in household*	9 (81.8%)	Ref	Ref
Prevalence by **Minimum Acceptable Diet Consumed**			
*Minimum Acceptable diet consumed*	72 (43.6%)	0.64	0.042
*No Acceptable Minimum Diet Consumed*	90 (54.9%)	Ref	Ref
Prevalence by **Ownership and Sleeping in Mosquito Nets**			
*Mosquito Net Present*	62 (48.4%)	Ref	Ref
*Mosquito Net Absent (Not Present)*	133 (55.9%)	1.57	0.009

### Childhood malnutrition (acute and chronic)

Global Acute Malnutrition (wasting) rate in general was 7.6% and this compares to 5% at the national level. Among the background variables disaggregated, month of child was significantly associated with risk of acute malnutrition (OR 1.16 95%CI 0.68–1.96 *p* < 0.001) with the 6 to 17 being the category with highest prevalence. Children situated with household size between 1 and 5 were significantly less likely to develop acute malnutrition (OR 0.32 95% CI 0.107–0.979) (Table 4).

**Table 4:** Prevalence of Global Acute Malnutrition (GAM) by background variables

Variable Disaggregation	n (%)	Odds Ratio (OR)	Sig. Level (P-value)
**GAM Rate**	44 (7.6%)		
Prevalence of GAM by **Communes**			
*Rumonge*	33 (7.60%)	0.933 Ref	0.894
*Vyanda*	12 (8.60%)		Ref
GAM by **Nutritional Status of caregiver**			
*Normal Nutritional*	18 (3.7%)	0.911	0.884
*Malnourished*	3 (4.0%)	Ref	Ref
Prevalence of GAM by **Household Hunger Scale**			
*No Hunger detected in household*	28 (6.90%)	0.36	0.35
*Moderate Hunger detected in household*	17 (10.90%)	0.44	0.46
*Severe Hunger detected in household*	9 (10.50%)	Ref	Ref
Prevalence of GAM by **Wealth Quintiles**			
*Highest*	6 (5.20%)	0.40	0.195
*Fourth*	6 (5.10%)	0.27	0.103
*Middle*	13 (10.80%)	1.07	0.897
*Second*	10 (8.90%)	Ref	Ref
*Lowest*	12 (10.40%)	0.26	0.103
Prevalence of GAM by **Household size**			
*1 to5*	17 (7.10%)	0.32	0.046
*6 to 10*	27 (8.60%)	Ref	Ref
*more than 10*	2 (9.50%)	1.79	0.457
Prevalence of GAM by **Age in Months**			
*6 to 17*	6 (12.70%)	1.16	< 0.001
*18 to 29*	13 (8.50%)	0.92	0.296
*30 to 41*	9 (6.80%)	Ref	Ref
*42 to 53*	2 (5.10%)	0.81	0.638
*54 to 59*	1 (4.50%)	1.02	0.332

The Global Chronic Malnutrition (stunting) rate was 45.8% (95%CI 42.5–49.1) which compares to 55% at the national level. Prevalence was higher among boys 48.4% (95% CI 43.7–53.1) than girls 43.3% (95% CI 38.8–47.9). Lack of Vitamin A administration 6 month before the survey was significantly associated with increased risk of childhood stunting (OR 1.76 95%CI 1.07–2.90). Households located in the poorest quintile were 1.8 times more likely to be chronically malnourished (Table 5).

**Table 5:** Prevalence of Global Chronic Malnutrition (GCM) by background variables

Variable Disaggregation	n (%)	Odds Ratio (OR)	Sig. Level (P-value)
**GCM Rate**	**266 (45.8%)**		
GCM by **Province**			
*Rumonge*	205 (46.7%)	1.63	0.120
*Vyanda*	54 (39.1%)	Ref	Ref
GCM by **Nutritional Status of caregiver**			
*Normal Nutritional*	221 (44.9%)	0.93	0.777
*Malnourished*	35 (46.7%)	Ref	Ref
GCM by **Household Hunger Scale**			
*No hunger detected in household*	163 (40.9%)	0.58	0.368
*Moderate Hunger detected in household*	90 (53.9%)	0.97	0.966
*Severe Hunger detected in household*	6 (54.5%)	Ref	Ref
GCM by **Wealth Quintiles**			
*Richest*	39 (33.9%)	0.710	0.212
*Fourth*	46 (40.0%)	0.92	0.764
*Middle*	62 (51.7%)	1.48	0.140
*Second*	47 (42.0%)	Ref	Ref
*Poorest*	65 (56.5%)	1.80	0.029
GCM rate by **Household Size**			
1 to5 people	107 (45.1%)	1.03	0.876
6 to 10 people	141 (44.5%)	Ref	Ref
more than 10 people	11 (47.8%)	1.14	0.755
GCM rate by **Vitamin A supplementation for children U5**			
*Yes Supplementation*	195 (42.8%)	Ref	Ref
*No Supplementation*	53 (57.0%)	1.76	0.027

### Childhood mortality

**Fig. 2:**
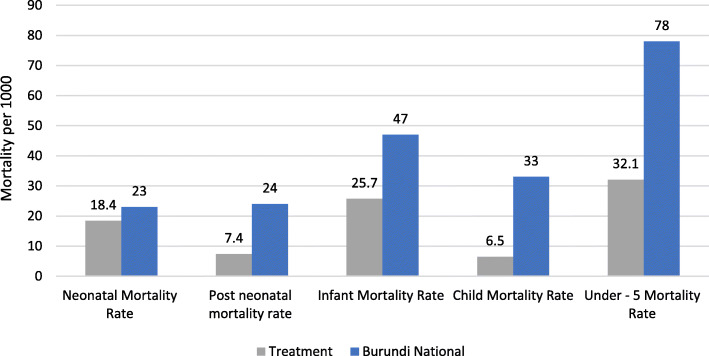
Comparative childhood mortality – Study area

In total, 2675 birth histories were collected in the program from which the mortality rates were computed. As illustrated in Fig. 2, neonatal mortality rate was 18.4 per 1000 live births compared to 23 per 1000 nationally. In the study area, neonatal mortality contributes to 57.3% to all under-5 mortality which compares to 25.7% nationally.

Post Neonatal Mortality Rate (PNNR) was 7.4 in the study area in comparison to 24 per 1000 nationally, representing 123.3% more nationally. Infant mortality was 58.6% higher at the national than in the study area (Fig. 2). Furthermore, child mortality 127.5% lower than the national figure.

## Discussion

Implementation of child health interventions is complex and therefore implementation approaches should be integrated with other sectors and multilevel of governance. This fundamental philosophy informed our program logic to implement an integrated intervention which priorities will be further determined by areas which showed strongest associations or increased risks with child mortality, stunting and wasting. For example, in our catchment area, most under-5 deaths happened in the first 28 days of life (57.3%). This particular finding has been reinforced by several studies and global reports. The World Health Organisation estimates that 2.5 million die in the first month of life, amounting to 47% of all under-five deaths [28]. Akseer et al. [29] further highlight that of these numbers, 73% die within the first 7 days and 36% on the first day of birth. Given that half of all these deaths occur within the first 48 h [29], strengthening all aspects of the health system to provide lifesaving integrated maternal and perinatal intrapartum care is critical. Our integrative intervention to be implemented takes account the incidence of low birth (who are at an increased risk of neonatal death) and will pay special focus to malnourished pregnant women who are even at increased risk. Inclusive in this integrated intervention is a US$ 20 million capital investment made by VHW for a hospital that will provide advanced diagnosis and treatment for malnourished children including those who suffer from malnutrition related complications.

Malnutrition emerged as one of the challenges to child health in the study area and for acute malnutrition, the risk of acute malnutrition was significantly higher among children aged 6 to 17 months. Earlier studies have confirmed this finding [30, 31] and Amsalu and Tigabu [30] further suggests that children in this age group are 10 times more likely to be acutely malnourished than cohorts in other age brackets. From field observations during the study, we found that especially for young and first-time mothers, there was an apparent difficulty in adhering to exclusive breastfeeding and appropriately introducing complementary foods after the initially six-month window. Both non-adherence to exclusive breastfeeding and inappropriate introduction of complementary foods have been directly linked to increased risk of acute malnutrition and mortality [32, 33]. Among the several community initiatives implemented to reduce incidence of acute malnutrition, our integrative model has a component of working with young and first-time mothers via the ‘parenting journey’ program. This program will pair up first time mothers with selected experienced and older women who will provide mentorship and support under the supervision of VHW. Evidence also point to increased risk of postpartum maternal depression among first time mothers [34], therefore, we hypothesize that this program will have improvements of maternal mental health as well.

Compared to an average of 33% in sub-Sahara Africa [35], chronic malnutrition manifested as stunted growth was high in our study area (47.8%) and was associated with low food diversity and food access. The mean food consumed out of eight food groups assessed was 2.5 with cassava being the most consumed food (87.0%). In most instances, cassava was consumed along with beans as the single, most important source of protein (76.6%). This pattern puts children at risk of inadequate nutrient intake [36] which also is also associated with increased risk of stunted growth. From informal discussions with community members, the preference of cassava emanated from a myriad of problems: lack of farmable land vis-à-vis increased household size, resistance of cassava to diseases, inadequate knowledge on diversified food cultivation and preparation. This complex situation will be tackled via creation of food security cooperatives whom will receive agricultural inputs and technical support for a year, after which, they will transition into an independent group. We hypothesize that the formation of cooperatives will mitigate the problem with land access for vulnerable households who are often times at risk of chronic malnutrition.

## Study limitations

Two limitations of the study which should be noted. First, although some variables were statistically associated with the outcomes under study, because of cross-sectional design we claim causality at this baseline stage. Another limitation is that apart from malnutrition that children were assessed for, results from all other indicators were acquired via verbal recall of caregivers. This could have resulted recall bias and either underestimated or overestimated findings, however, earlier studies have established the validity and reliability of this methodology [22, 23]. Moreover, we initially piloted and contextualised the survey instrument for relevance to the study setting. Also to reduce the possibility of recall bias, samples of supplements and medicines were presented to the caregiver for identification.

## Conclusion

Interventions to improve neonatal, infant and child health are complex and as such, its success will be dependent on the extent to which it is integrated to address underlying the causes. Lessons from VHW’s previous program implementation highlights the importance of active community engagement in building trust and solidarity to promote allopathic-based care offered by community health workers and the health facilities. This implies that community members and actors should play key role in every stage of health service planning, program implementation and evaluation.

Equally important in improving under-five health is strengthening all the six components of a health system to provide quality and compassionate care for all. Especially in fragile settings, creating an optimal model of healthcare delivery and building sustainable systems to train the next generation of health workforce is a giant step. This process requires wide stakeholder engagement with government at the centre with a deep focus on sustainability post-intervention.

## Data Availability

The data used for analysis in this manuscript are available from the Research, Monitoring and Evaluation Department of VHW. Data is restricted but available from corresponding author upon reasonable request.

## Abbreviations

CMR: Child mortality rate
DHS: Demographic Health Survey
ENA: Emergency Nutrition Assessment
IMR: Infant mortality rate
LBW: Low Birth Weight
MUAC: Mid-Upper Arm Circumference
NNMR: Neonatal mortality rate
OR: Odds Ratio
PNMR: Post-neonatal mortality rate
PPS: Probability Proportional to Size
SMART: Standardized Monitoring and Assessment of Relief and Transitions
SPSS: Statistical Package for the Social Sciences
U5MR: Under-5 mortality rate
UNICEF: United Nations Children’s Fund
VHW: Village Health Works
WHO: World Health Organisation
